# Notes on Preparations for the Sick

**Published:** 1892-03

**Authors:** 


### Notes on Preparations for the Sick.

**Peptonoids Iron and Wine. Maltine with Cascara Sagrada. Maltine with Lime. Maltine with Pepsin and Pancreatine. Tablets of Cascara Sagrada with Chocolate.**—Maltine Manufacturing Company, London.

The preparations of the Maltine Manufacturing Company are for the most part well known, but deserve to be more so. The peptonoid powder, and its cordial-like solutions, in combination with coca, or with pyrophosphate of iron in sherry, are invaluable medicines where the maintenance of nutrition is of primary importance.

The use of maltine as a vehicle for nauseous drugs is being well recognised. The various combinations have an infinitude of utility, are well devised, and have been abundantly proved to be efficient.

---

**Liquor Carnis. Beef-Tea Jelly. Malto-Carnis. Malto-Carnis and Cocoa. Carnis Suppositories.** —Liquor Carnis Company, London.

The uncooked juice of beef in an agreeable form, always ready for use, and yet capable of being preserved indefinitely, must be invaluable in the sick-room.

The one drawback is the sweetness of liquor carnis. An attempt has been made to overcome this by flavouring with celery, and the fluid may be given disguised in many ways. It may be mixed with warm fluids, soups, coffee, cocoa, arrowroot, &c., or it may be taken cold with port wine, diluted spirit, or lemon squash—in either case without loss of its nutritive value. It is also an ideal fluid for rectal administration where nutrient enemata are required. It contains an amount of soluble albumin equal to 1.5 per cent. as estimated by Esbach's albuminimeter. The term "fluid muscle," which its makers sometimes use in describing it, expresses the value which is set upon it.

Malto-Carnis contains two-thirds of the liquor in combination with extract of malt and cocoa. It is an ideal food for use in cases of debility from any cause, and as it contains no sugar it may be added to the restricted list of foods suitable for diabetics. Taken in hot milk, it forms a most nutritious draught, and many patients who have become tired of everything else have been grateful for its introduction.

The Suppositories are prepared by the addition of a colloid medium, which melts readily at the temperature of the body, and each of them contains the equivalent of two drachms of the liquor. It has been found by experiment that the albumin of expressed meat juice is absorbed to nearly the same extent as complete peptone.

---

**Hartmann's Wood Wool Wadding. Wood Wool Tissue. Hygienic Wood Wool Diapers. Wood Wool Gonorrhœa Bags. Wood Wool Vaccination Pads. Wood Wool Antiseptic Sponge.**— THE SANITARY WOOD WOOL COMPANY, London.

Hartmann's Wood Wool is the most absorbent dressing known. The principal objection to its use is the want of cohesion of its particles, and this objection is overcome in the various useful preparations now made by the Sanitary Wood Wool Company.

The *Wood Wool Wadding* is as easily manipulated as cotton wool, and is much more absorbent. It is prepared either plain or medicated with various antiseptics, such as corrosive sublimate, carbolic acid, salicylic acid, &c., and in these forms it is probably the most perfect dressing for wounds ever invented.

*Wood Wool Tissue* is prepared in sheets, and consists of a layer of wood wool between two layers of muslin; it is very absorbent and easily applied.

*Wood Wool Diapers* are invaluable to ladies while travelling or after parturition. The ordinary linen napkin is so slightly absorbent that it serves to retain secretions in the vagina, but these diapers are comfortable, clean, and healthful. The same remarks apply to the *Wood Wool Gonorrhœa Bags*, which are muslin bags lined with wood wool, rendered antiseptic by corrosive sublimate, and furnished with two tapes, by means of which they may be fastened around the waist. They are very cleanly, preventing any soiling of the clothes by the discharge, and thereby diminish the risk of infecting other mucous surfaces.

The *Vaccination Pads* are prepared with a layer of wood wool to go next the vesicles and a layer of muslin over, with tapes for fastening in position. They would be invaluable for the very sore arms occasionally met with after vaccination.

*Wood Wool Antiseptic Sponges* are calculated to displace entirely the old-fashioned sponges used during operations. In the well-regulated operation room one now hardly ever sees an ordinary sponge in use, and these wood wool sponges are simple, clean, and cheap, so that they can be freely used, and burnt afterwards.

---

**Dermatol.**—Burroughs, Wellcome & Co., London.

Dermatol is intended for use in some skin affections and as a general substitute for iodoform. It is insoluble in water, very stable, and quite odourless, which gives it an immense superiority over iodoform. It seems to have strong antiseptic properties; for granulation wounds and surfaces discharging sloughs keep quite healthy and free from putrefaction under its use. For intertrigo and skin excoriations it should be used mixed with starch, and then it forms a most soothing application.

---

Mr. F. J. Rebman, of 40 Berners Street, London, who is the agent for the Animal Lymph cultivated from calf to calf at the Institute of Dr. Pissin, Berlin, has sent us specimens of the two kinds which are supplied, with each of which we have produced excellent vesicles. One of the kinds is emulsified, and is recommended in those cases where the lymph has to be kept for some time.

